# Shear Wave Elastography in Diagnosing Secondary Hyperparathyroidism

**DOI:** 10.3390/diagnostics9040213

**Published:** 2019-12-05

**Authors:** Laura Cotoi, Florin Borcan, Ioan Sporea, Dana Amzar, Oana Schiller, Adalbert Schiller, Cristina Adriana Dehelean, Gheorghe Nicusor Pop, Dana Stoian

**Affiliations:** 1 PhD School Department, “Victor Babes” University of Medicine and Pharmacy Timisoara, 2nd Eftimie Murgu Square, 300041 Timisoara, Romania; cotoi.laura@umft.ro; 2 Analytical Chem. and Toxicology Department, “Victor Babes” University of Medicine and Pharmacy Timisoara, 2nd Eftimie Murgu Square, 300041 Timisoara, Romania; cadehelean@umft.ro; 3 Internal Medicine 2nd Department, “Victor Babes” University of Medicine and Pharmacy Timisoara, 2nd Eftimie Murgu Square, 300041 Timisoara, Romania; isporea@umft.ro (I.S.); schiller.adalbert@gmail.com (A.S.); 4 Endocrinology Department, “Victor Babes” University of Medicine and Pharmacy Timisoara, 2nd Eftimie Murgu Square, 300041 Timisoara, Romania; stoian.dana@umft.ro; 5 Dialysis Medical Center B Braun Avitum, 636 Remetea Mare, 307350 Timisoara, Romania; oana.schiller@bbraun.com; 6 Centre for Modelling Biological Systems and Data Analysis, Department of Functional Sciences, “Victor Babes” University of Medicine and Pharmacy Timisoara, 2nd Eftimie Murgu Square, 300041 Timisoara, Romania; pop.nicusor@umft.ro

**Keywords:** elastography, parathyroid, secondary hyperparathyroidism, shear wave elastography, ultrasonography

## Abstract

This study evaluates the diagnostic value of two-dimensional shear wave elastography (2 D-SWE) technique in the evaluation of hyperplastic parathyroid glands in cases with secondary and tertiary hyperparathyroidism. A total of 59 patients (end-stage renal disease, under supplemental dialysis program) with visible parathyroid hyperplastic glands on ultrasound, confirmed by biochemical assay and scintigraphy, were enrolled; they were examined on grayscale ultrasound and 2 D shear wave elastography. We determined the elasticity index (EI) in the parathyroid gland, thyroid parenchyma and surrounding muscles, and the elasticity ratio of hyperplastic parathyroid glands compared to muscle, specifically sternocleidomastoid muscle. Patients presented fibrocystic bone disease with secondary hyperparathyroidism induced by end-stage chronic kidney disease; being on prolonged chronic dialysis therapy, they had positive sestamibi scintigraphy and high values of serum parathormone (1141.04 pg/mL). Nodules placed posterior to the thyroid capsule that were cystic, had a hypoechoic aspect, and were homogenous with an independent afferent artery were found. Mean EI in the parathyroid gland was 7.83 kPa, the median value in thyroid parenchyma was 13.76 kPa, and mean muscle EI value was 15.78 kPa. The observed mean parathyroid/muscle SWE ratio was 0.5356 and the value for parathyroid/normal thyroid parenchyma was 0.5995. Using receiver operating characteristic (ROC) analysis, we found that EI below 9.74 kPa correctly identifies parathyroid tissue, with a sensitivity of 94.8%, specificity of 90.7%, and accuracy of 92.26% when compared to normal thyroid tissue. Compared with the muscle tissue, we identified that EI below 9.98 kPa has a sensitivity, specificity, and accuracy of 93.8%, 90.7%, and 91.75%, respectively. Ultrasound elastography is a helpful tool in identifying parathyroid hyperplasia in patients with chronic kidney disease. A cutoff value of 9.98 kPa can be used in 2 D-SWE for accurate diagnosis of parathyroid disease.

## 1. Introduction

Secondary hyperparathyroidism (sHPT) is a prevailing complication of chronic kidney disease (CKD) caused by the disturbance of calcium, phosphate, and vitamin D, with high concentrations of serum parathormone (PTH) leading to high rates of cardiovascular and bone disease [1,2]. 

The incidence and prevalence of chronic kidney disease, including kidney failure requiring renal replacement therapies (RRT) is rising in Europe. Incidence in Europe is estimated at 119 per million population (pmp), the highest incidence is cited in the Czech Republic (232 pmp). Prevalence is reported at 801 per million population, with the highest rate reported in Portugal (1824 pmp) [3]. In the United States, the prevalence of CKD is estimated at around 15% of the total population, and over 700,000 people are registered in the end-stage renal disease (ESRD) program, with an incidence of over 120,000 cases every year [4]. 

The Kidney Disease: Improving Global Outcomes (KDIGO) guidelines recommend that screening and management of sHPT should be proposed to all patients starting CKD stage 3 [1,5]. The prevalence in Europe of sHPT among patients with requiring RRT is estimated at 43.8% in France, 46.8% in Russia, and 42.9% in the United Kingdom. United States has a prevalence of 54% [6]. 

The pathogenesis of sHPT is multifactorial and complex, driven by hypocalcemia, low levels of vitamin D, hyperphosphatemia, and high concentration of fibroblast growth factor 23 (FGF 23). The discovery of the phosphaturic hormone FGF 23 and counter-regulatory hormone for vitamin D remodel the traditional pathophysiological scheme of sHPT. The new pathogenesis of sHPT includes in the first steps a renal decrease in renal phosphate excretion (due to loss of kidney mass) and an increase in FGF 23 secretion from the osteoblasts. The high concentrations of FGF 23 inhibit phosphate reabsorption in the kidney and inhibit vitamin D concentrations [7,8].

The Kidney Disease: Improving Global Outcomes (KDIGO) guidelines recommend that all patients should be screened and receive treatment for secondary hyperparathyroidism with CKD stage 3 (estimated GFR < 60 mL/min). The management of sHPT is a stepwise technique that aims to optimize serum concentrations of calcium and phosphorus, combining low phosphorus diet with pharmacological treatment [1,5,9,10]. 

KDIGO guidelines for CKD stage 5 (estimated GFR < 15 mL/min) suggest that serum PTH concentrations should be maintained between two to nine times the upper normal level for patients in dialysis [5]. 

The primary treatment option recommended for patients with secondary hyperparathyroidism due to chronic kidney disease, besides dietary restrictions, is medical treatment. It includes phosphate binders, calcium supplements, active vitamin D analogues, and calcimimetics, to help reduce the PTH serum level and restore the calcium–phosphate homeostasis, although some patients still require parathyroidectomy to confront the complications of the disease [1,5,11]. Regarding the PTH target according to the KDIGO guidelines, in patients with CKD G5 D on dialysis the suggested PTH levels are approximately two to nine times the upper normal [12].

KDIGO guidelines recommend parathyroidectomy in patients with CKD G3 a to G5 D with severe hyperparathyroidism, refractory to medical or pharmacological treatment, in order to prevent cardiovascular complications and ectopic calcifications and to improve quality of life [5,13]. Secondary hyperparathyroidism is defined as a PTH two to nine times the upper normal value of the normal values of the healthy population (15–65 or 15–46 pg/mL [14]). Chronic kidney disease–mineral bone disorder (CKD-MBD) currently has a broader definition, including: Abnormalities of calcium, phosphorus, PTH or vitamin D.; abnormalities in bone turnover, mineralization or strength [15]; and even isolated hyperphosphatemia or, in patients with calciphylaxia, serum PTH concentration above 500 mg/dl [16].

Literature reports suggest that a serum PTH concentration above 800 pg/mL, accompanied with sustained hypercalcemia (serum calcium higher than 10.4 mg/dl) and hyperphosphatemia, despite medical treatment, is an absolute indication for parathyroidectomy, especially in patients awaiting renal transplantation [13,17]. Patients with severe symptomatic bone disease, pruritus, myopathy, and calciphylaxia are also candidates for parathyroidectomy. Asymptomatic patients with serum PTH concentrations higher than 1000 pg/mL, refractory to medical and pharmacological treatment, are also referred to surgery. Literature studies support preoperative localization of parathyroid glands (ultrasound) in sHPT for primary parathyroidectomy and other imaging modalities in re-operative renal hyperparathyroidism [13].

Considering the treatment options, a correct identification of the location of the parathyroid glands is mandatory before referring to surgery. 

Current presurgical localization options are conventional ultrasonography, scintigraphy, computed tomography (CT), and magnetic resonance imaging (MRI). The described sensitivity in localization of the parathyroids were: 48%–92% for scintigraphy, in cases without previous surgery [17]; 46%–80% for computed tomography; and 33%–71% for magnetic resonance [18]. Different studies have showed high-resolution ultrasound sensitivity of 33%–66% [18], 73% [19] with specificity of 90%, and accuracy of 81% [20,21]. There is no perfect method for localizing parathyroid hyperplasia in sHPT, and the sensibility and specificity increase when using multiple or complementary localization methods [21].

Ultrasonography (US) is the most accessible imaging technique for the localization of parathyroid disease. Ultrasonography is widely used, and it is valued for its reproducibility, ease of use, noninvasiveness, high resolution, no exposure to X-rays, no need for contrast agents, and real-time investigation and because it is harmless to children and pregnant women [22]. As a complementary technique to ultrasound, elastography gives qualitative and quantitative information about tissue stiffness. Labeled as “palpation imaging”, elastography has been successfully validated in many clinical areas, such as breast tumor characterization [23], hepatic fibrosis staging [24,25] and thyroid nodules [26], testicular cancer [27], and there have been studies conducted in primary hyperparathyroidism [28,29,30]. 

The coexistence of thyroid disorders, hypothyroidism, and nodular goiter make parathyroid ultrasonography a difficult task in identifying parathyroid glands in these patients. In cases of RRT treatment, the thyroid pathology is more frequent than in the general population [31,32,33,34,35].

The correct identification of parathyroid lesions, in cases with coexistence of thyroid lesions, is still a task for clinicians. Shear wave elastography is used to quantify tissue elasticity by evaluating shear wave attenuation. Shear waves are vibrations induced by acoustic radiation force through a focused ultrasound beam, making it a more operator-independent technique [36,37,38]. By analyzing the particle displacements with an ultrafast ultrasound acquisition sequence, quantitative measurements of tissue elasticity can be obtained [36]. Evaluation of the elasticity characteristics of the tissue can help in identifying special types of tissues [39]

Shear wave elastography (Aixplorer Super Sonic) overlays the elastographic image on the B-mode, providing a real-time map of elasticity. The operator should also pay attention not to apply any kind of pressure on the probe [40]. The region of interest (ROI) is positioned on the area of interest (in our case hyperplastic parathyroid gland) and in the surrounding thyroid tissue and surrounding muscle. For each of the ROIs, the ultrasound software calculates the mean, minimum, and maximum stiffness, as well as the standard deviation. It can also provide the elasticity–shear wave ratio (SWR) between two ROIs. There are no elastography scale recommendations for parathyroid evaluation, so thyroid elastography scale can be used and set at 100 kPa [41].

The objective of this prospective study was to determine, using 2 D shear wave elastography, the elastographic characteristics of hyperplastic parathyroid glands in patients with chronic kidney disease and determine whether the technique adds diagnostic and localization information of these glands. Identifying a threshold value suggestive for parathyroid tissue will add diagnostic value in presurgical identification of parathyroid hyperplastic glands, especially in cases with intrathyroid parathyroid or in cases with associate thyroid nodular lesions.

## 2. Materials and Methods

This is a prospective study was conducted from May 2019 to July 2019 in the Dialysis Medical Center B Braun Avitum Timisoara, Romania. All patients over 18 years of age with stage 5 CKD were under hemodialysis therapy. All patients received hemodialysis three times per week. 

From the total of 120 cases present in the treatment center, 59 patients presented parathyroid disorders by clinical, biochemical assessment and scintigraphy scan or MRI scan. The study was approved by the Ethics Committee of the Dialysis Center, and all patients signed a written informed consent. The study was in accordance with the Ethics Code of the World Medical Association.

### 2.1. Inclusion Criteria

All patients were adults with confirmed end-stage renal disease, on RRT (renal replacement therapy), who presented secondary hyperparathyroidism for more than six months prior to the ultrasound evaluation. We included patients with confirmed secondary hyperparathyroidism by biochemical assay that was confirmed by scintigraphic and/or MRI evaluation. We also included patients with previous subtotal parathyroidectomy, which presented parathyroid enlargement on ultrasound. The final analysis was made on total identified parathyroid glands, regardless if one or more hyperplastic glands were identified in each patient. 

### 2.2. Exclusion Criteria

Ectopic parathyroid glands, diagnosed by means of MIBI scintigraphy, were excluded. Secondary cases of hyperparathyroidism that underwent total parathyroidectomy, with secondary adynamic bone disease, were also not considered in the evaluation.

### 2.3. Conventional Ultrasound

Conventional B-mode parathyroid ultrasound was performed in all cases on Aixplorer Mach 30 (SuperSonic Imagine, France) using a high-resolution linear transducer of 15–4 MHz, with the patient resting in supine position with regular breathing, applying an adequate amount of ultrasound gel.

Using grayscale ultrasound (US), we evaluated the following parameters: Thyroid dimensions (two dimensions in transverse scan and one dimension in longitudinal), thyroid volume, parathyroid hyperplasia dimensions, parathyroid volume, shape, and echogenicity. Doppler US was performed in order to observe the presence of vascular pattern on the parathyroid glands. All patients were clinically evaluated and with ultrasonography by two practitioners, one with over 15 years of experience in thyroid, parathyroid, and neck ultrasound.

### 2.4. Elastography Exam

After performing conventional ultrasound, two-dimensional shear wave elastography (2 D-SWE) was performed with Aixplorer system (SuperSonic Imagine, France) using two high-resolution multifrequency linear transducers of 15–4 and 18–5 MHz. We used each probe for each case, but we analyzed the best images—the more profound lesions needed the 15–4 MHz probe, with the 18–5 MHZ offering better resolution. Patients remained in supine position, with hyperextension of the neck, the examiner maintained precise adherence of the probe to the examination area without applying any manual compression, permitting the transducer to automatically induce vibrations in the tissue and avoid any movements while waiting for the image stabilization.

**Figure 1:**
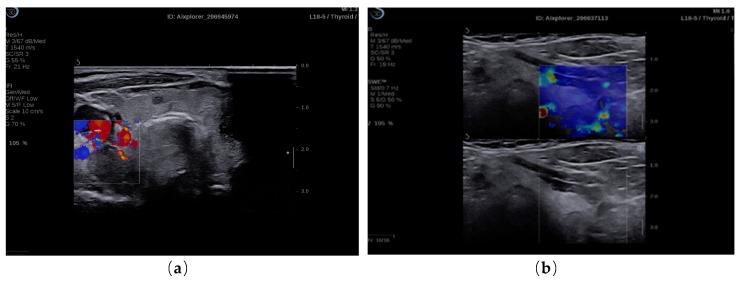
(**a**) 2 B-mode ultrasound evaluation of parathyroid hyperplasia, visualizing parathyroid hyperplasia in color dopple-r mode; (**b**) elastogram overlapping B-mode image of parathyroid hyperplasia, color map of tissue elasticity.

Real-time elastograms overlap on B-mode image determining tissue stiffness (Figure 1). 

Qualitative evaluation of 2 D-SWE shows the standard elasticity maps, offered standard by Supersonic (blue—soft tissue, red—stiff tissue), in the entire ROI. The size of ROI was big enough in order to comprise the needed sites to evaluate. 

The quantitative evaluation was preferred, with measurement of elasticity parameters. Taking in account that there are no scale settings for parathyroid evaluation, thyroid default settings were used (range 0–100 kPa).

The summary of tissue elasticity properties are automatically displayed by the machine software, after placing the quantification box (Q-box) on the lesion needed to be measured. The size of the Q-box was identical to the size of the parathyroid observed adenoma. The obtained elasticity parameters by our 2 D-SWE machine were: Maximum stiffness value (SWE-max), minimum stiffness value (SWE-min), mean stiffness value (SWE-mean) and standard deviation (SWE-SD). For each parathyroid gland, we performed five consecutive measurements. All shear wave measurements were recorded numerically (expressed in kPa).

**Figure 2:**
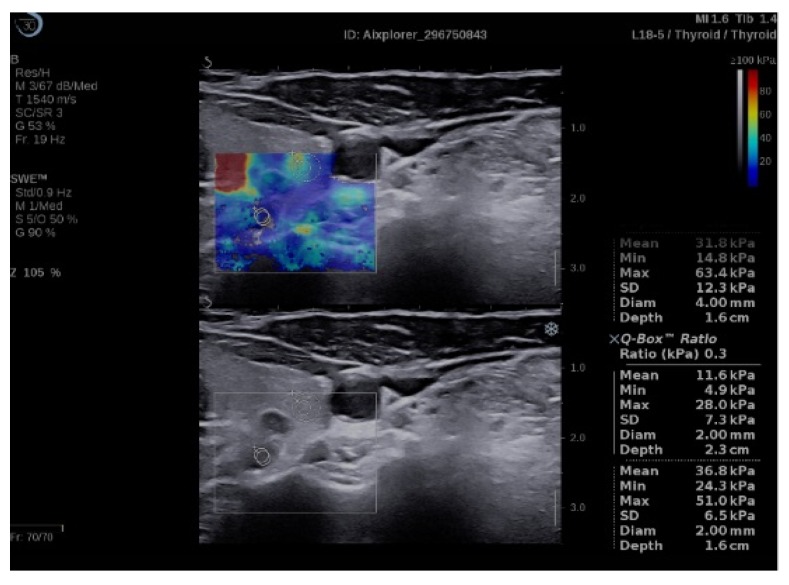
Quantification box (Q-box) ratio of parathyroid hyperplasia with thyroid tissue.

**Figure 3:**
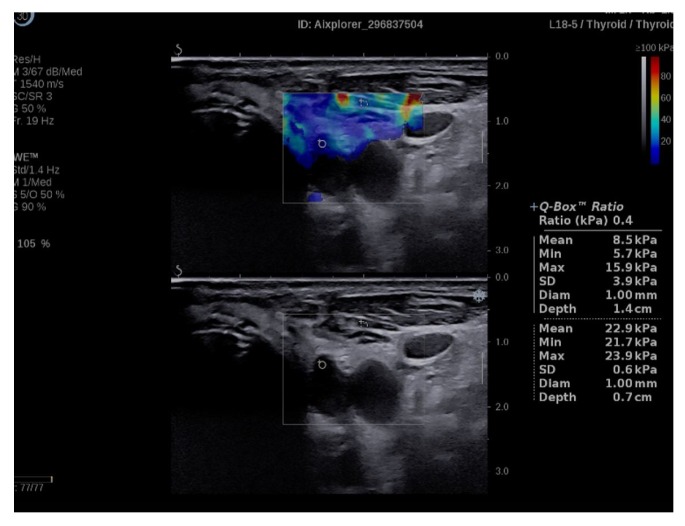
Q-box ratio of parathyroid hyperplasia with muscle tissue.

Q-box ratio allows the comparison of the stiffness of two different areas present in the ROI, resulting in a numerical parameter, SWE-ratio (SWR) [42]. We compared the elasticity index of the parathyroid adenoma, using the initial Q-box, with the elasticity index of the healthy adjacent thyroid tissue, using same sized Q-box (Figure 2) and also with the elasticity index of the same side sternocleidomastoid muscle (in longitudinal plane section) (Figure 3). We obtained comparative ratio also with muscle, for the practical future use in cases with no preserved/normal thyroid tissue. 

### 2.5. External Observer Evaluation

The ultrasound and elastographic images were additionally reviewed by two clinicians, one with over 15 years of experience in ultrasonography and elastography in thyroid and neck ultrasound. They were blinded about the technetium sestamibi scintigraphy prior to the ultrasound evaluation. 

### 2.6. Biochemical Assay

Complete clinical and biochemical profile was obtained for each patient. Before receiving hemodialysis, approximately 10 mL of blood was collected, centrifuged, stored at 4 °C, and used for biochemical analyses within 1 h after collection. Serum values of blood urea nitrogen, creatinine, glucose, total cholesterol, triglyceride, total calcium, and phosphorus were examined by an auto analyzer (Dimension RxL Max Integrated Chemistry System, Siemens, mass spectrophotometry method). 

The adequacy of hemodialysis was calculated as fractional clearance index for urea (Kt/V) and urea reduction ratio by using single compartment dialysis urea kinetic model. The following measurements were taken in consideration: Calcium (reference range 8.5–10.2 mg/dL), mass spectrophotometry method; PTH (15–65 pg/mL), immunochemistry with enzyme chemiluminescence immunoassay (ECLIA); vitamin D (30–100 ng/mL), immunochemistry with ECLIA; and the index of urea Kt/V was directly calculated for each patient. We did not consider in this study other complications such as bone disease or cardiovascular risk.

### 2.7. Technetium-99 Sestamibi Scintigraphy Scan

All 59 cases enrolled in the study were patients with at least one hyperplastic parathyroid gland with a diameter greater than 0.5 cm, confirmed by clinical and biochemical evaluation and location by Sestamibi parathyroid scintigraphy (MIBI). The scintigraphy scans were analyzed by nuclear medicine specialists, who did not know about the ultrasound scan.

### 2.8. Statistical Analysis

Statistical analysis was performed on the total number of 97 described parathyroid glands in 59 patients. Continuous variables were presented as mean and standard deviation (SD), and categorical variables were presented as frequency and percentages. Bias-corrected and accelerated bootstrap interval (1000 bootstrap samples) was used to calculate the 95% confidence interval limits. We performed descriptive and inferential statistics analysis to summarize the characteristics of the study population. The results of the Shapiro–Wilk normality test showed a Gaussian distribution.

To highlight the parathyroid elasticity, we analyzed the strength of a linear relationship between the elastographic measures obtained using SWE and the baseline values using Pearson correlation. To compare patients’ characteristics in Table 1, we used the *T*-test for numerical variables. For comparing the SWE values of parathyroid, thyroid, and muscle, we used the ANOVA test, followed by a post-hoc analysis with Tukey test. Characteristic (ROC) curve was employed to illustrate the identification ability, and the thresholds to discriminate between the parathyroid and thyroid respectively muscle were determined with Youden’s index. A *p* value of <0.05 was considered to indicate a statistically significant difference. Data analysis was performed using SPSS 26 (Statistical Package for the Social Sciences, Chicago, IL, USA). 

The study was approved by the Ethics Committee of our hospital, and all patients signed a written consent.

## 3. Results

We evaluated 59 patients (male to female ratio 27:32) with mean age of 56.95 ± 10.92, mostly above 65 years old, with confirmed CKD stage 5, registered on ERSD (end-stage renal disease) program, on RRT (renal replacement therapy). Conventional ultrasound examination and 2 D-SWE elastography was performed. Baseline characteristics of the study group are presented in Table 1.

### 3.1. SWE Results

A total number of 97 hyperplastic parathyroid glands were studied. Five measurements were made for each parathyroid gland, and comparison was made with normal thyroid tissue and muscle tissue. We noted our SWE result in Table 2, considering the minimum SWE value, maximum SWE value, and the mean SWE value for each analyzed tissue.

**Figure 4:**
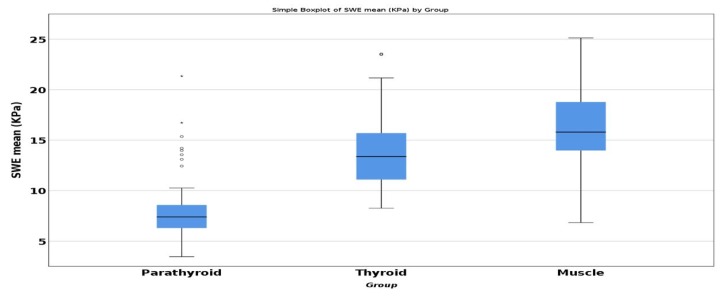
Comparison of mean SWE between parathyroid, thyroid, and muscle.

ANOVA test demonstrated a statistically significant difference for SWE-mean of parathyroid, thyroid, and muscle. Post-hoc analysis with Tukey test was conducted. Referring to parathyroid SWE, we observed statistically significant difference compared with the healthy thyroid SWE (−5.945, *p* < 0.001) and with the same-sided muscle (−7.953, *p* < 0.001) (Figure 4).

Based on ultrasound and scintigraphic results, a receiver operating characteristic (ROC) curve was used to evaluate the discriminative power of quantitative elastography measured parameters: SWE-max, SWE-min, SWE-mean, and SWE ratio.

The results summarizing AUROC, sensitivity and specificity for each quantitative parameter are presented in Table 3. The best sensitivity and specificity are observed for SWE-mean value.

By analyzing the parameters given by the ultrasound machine, we have found that the best accuracy is by analyzing the mean SWE value with a value of 92.26%, followed by the min SWE value with an accuracy of 90.7%. Min SWE and max SWE values could also be useful tools whenever we find borderline mean SWE values, helping to discriminate structures and positively identify parathyroid glands. 

Using muscle as standard, the area under curve (AUC) for parathyroid mean SWE was 0.949, 95% CI (0.917; 0.980). 

**Figure 5:**
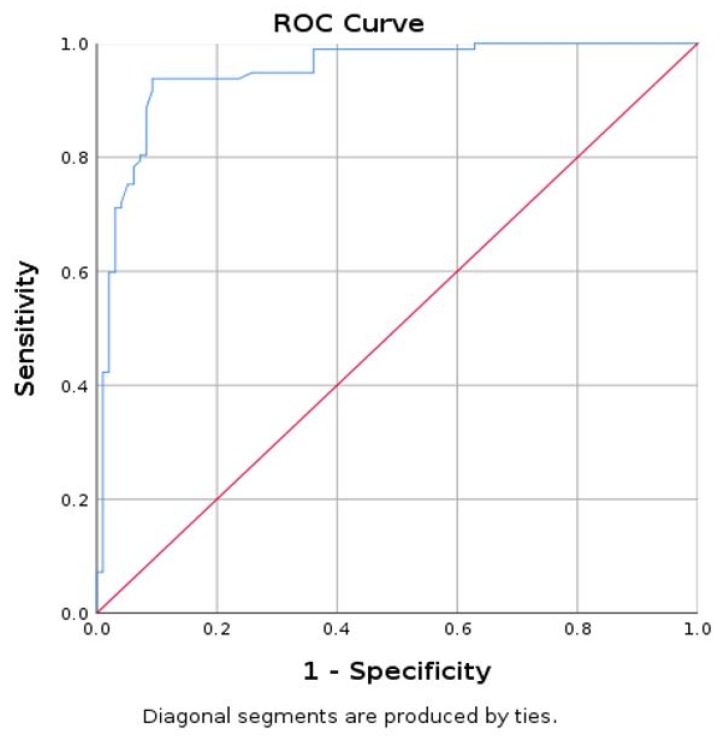
Area under curve (AUC) for prediction of parathyroid using mean SWE-mean for muscle (parathyroid as reference tissue).

A mean SWE value lower than 9.98 kPa has specificity of 90.7%, sensitivity of 93.8%, and accuracy of 91.75% in identifying parathyroid adenoma using muscle as reference (Figure 5).

**Figure 6:**
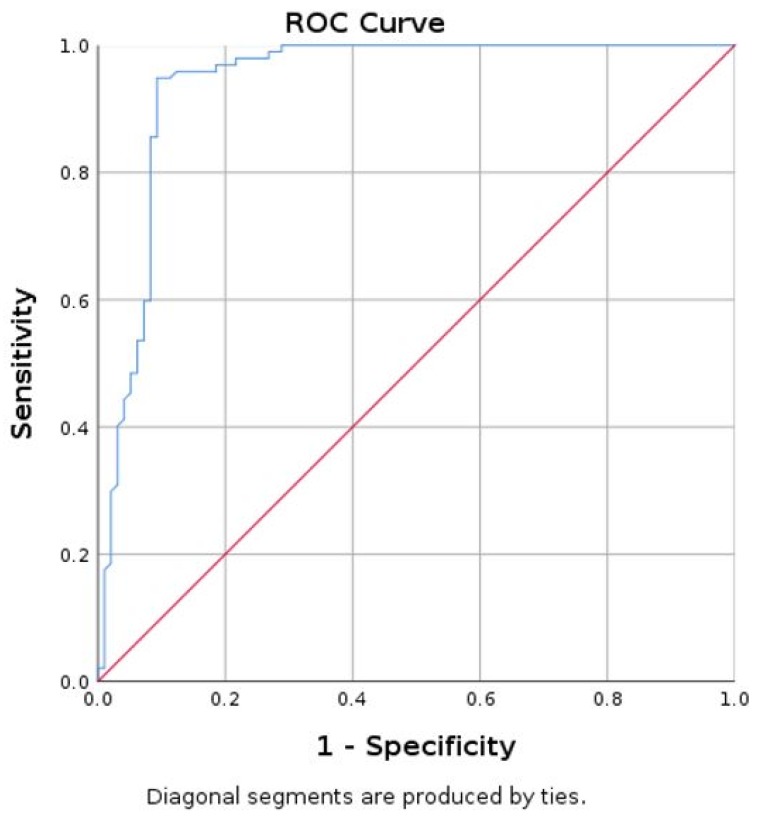
Area under curve (AUC) for prediction of parathyroid using mean SWE-mean for thyroid (parathyroid as reference tissue).

Using thyroid tissue as standard, the area under curve (AUC) for parathyroid mean SWE was 0.940 with 95% CI (0.903; 0.978) (Figure 6). 

A value of mean SWE lower than 9.74 kPa has a specificity of 90.7%, sensitivity of 94.8%, and accuracy of 92.26% in differentiating parathyroid tissue, in reference to thyroid tissue.

In respect to the shear wave ratio (SWR) obtained in our study group, we illustrate our results in Table 4. The SWR obtained for parathyroid hyperplastic tissue in comparison with the thyroid tissue was slightly higher than the SWR for parathyroid tissue with the muscle tissue. However, the ratio values were close to each other.

**Figure 7:**
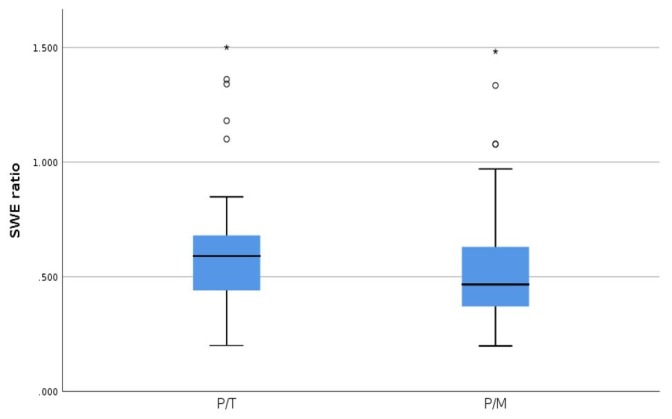
SWE ratio parathyroid tissue/thyroid tissue (P/T) and SWE ratio parathyroid tissue/muscle tissue (P/M).

**Figure 8:**
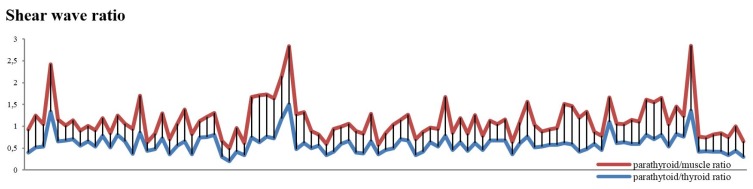
SWE ratio for parathyroid/muscle and parathyroid/thyroid values—compared values using stacked chart line.

When comparing the two SWE ratios, we obtained significant differences, using statistic test *T*, between the two groups (*p* = 0.001); however, ROC curve does not apply (Figure 7) because we cannot make a clear demarcation between the two, as the values are so close to one another (Figure 8).

Even if there are significant differences using SWE ratio for determining parathyroid hyperplastic tissue, there is an insufficient delimitation between the two reports (parathyroid/thyroid; parathyroid/muscle). The most reliable method to differentiate the hyperplastic parathyroid tissue from the other surrounding tissues is using SWE-mean.

### 3.2. Correlation between Shear Wave Elastography and Biochemical Assay

We compared the mean value obtained using shear wave elastography technique with the total duration of dialysis, parathyroid volume, serum PTH, phosphorus, and vitamin D concentrations.

When comparing the mean SWE for parathyroid tissue-obtained values with the total dialysis time, we did not find a significant correlation (*r* = 0.083, *p* = 0.420). We compared the mean SWE values for parathyroid with the PTH serum concentrations and did not find any correlation (*r* = 0.188, *p* = 0.66). 

**Figure 9:**
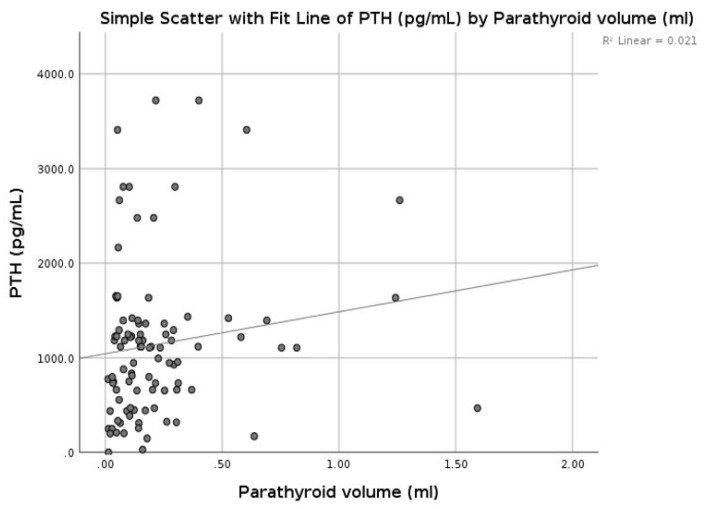
Correlation between parathormone (PTH) and parathyroid volume.

In patients with CKD, on RRT therapy, chronic stimulation of the parathyroid glands triggers diffuse polyclonal hyperplasia [1]. We compared the parathyroid volumes from our patients with the PTH serum concentration and did not find any significant correlation (*r* = 0.145, *p* = 0.158) between the two entities (Figure 9).

We did not observe any correlation between the mean parathyroid SWE value and parathyroid volume (*r* = 0.010, *p* = 0.922), or between the Kt/V coefficient (*r* = 0.181, *p* = 0.77) with vitamin D concentrations (*r* = 0.018, *p* = 0.857). 

Literature studies have suggested that parathyroid volume in sHPT has a positive correlation with the phosphorus levels. We compared our parathyroid volumes with phosphorus levels, but no significant correlation was found (*r* = 0.126, *p* = 0.221). Mean parathyroid SWE measurements compared with phosphorus concentrations do not correlate (*r* = 0.049, *p* = 0.635).

## 4. Discussion

In this prospective study, we determined the elastographic features using shear wave elastography of confirmed parathyroid hyperplasia secondary to end-stage renal disease and compared it to the elastographic features of surrounding thyroid and sternocleidomastoid muscle. The study included hyperplastic parathyroid glands that were initially localized in conventional B-mode ultrasonography and confirmed using parathyroid technetium sestamibi scintigraphy. Our study included patients from a single dialysis center. 

The role of ultrasonography and elastography is not defined in the international guidelines for secondary hyperparathyroidism. There is no “when and why” applied for parathyroid ultrasound in the management of real secondary hyperparathyroidism. Because the thyroid nodular disease is very frequent in ERDS cases [31,32,33,34,35] and the discriminative diagnostic is mandatory, identification and localization of hyperplastic parathyroid is mandatory.

The role of elastographic measurements in the parathyroid field is advancing, as several studies on the elasticity of parathyroid adenomas have been conducted and have shown that elastography is a helpful technique in differentiating parathyroid pathology [28,43,44,45]. 

In our study, tissue stiffness of parathyroid hyperplasia measured by SWE-mean provided quantitative information that may be useful in diagnosing and treating parathyroid hyperplasia and preventing tertiary hyperparathyroidism. The mean (±SD) SWE was 7.83 ± 2.94 kPa for all parathyroid glands enrolled in the study. When comparing the elasticity index of the hyperplastic parathyroid glands with the thyroid parenchyma mean SWE (13.780 ± 4.039 kPa) and surrounding muscle (15.788 ± 4.409 kPa), the elasticity of parathyroid hyperplasia was significantly lower than both thyroid and muscle tissue. The cut-off value of below 9.74 kPa had a sensitivity and specificity of 94% and 90%, respectively, when identifying parathyroid tissue in comparison with thyroid tissue. A value of below 9.98 kPa had a specificity and sensitivity of 90% and 93%, respectively, when identifying parathyroid tissue in comparison with muscle tissue.

To our knowledge, this is the first elastographic study performed with shear wave elastography on patients with end-stage renal disease on RRT and hemodialysis. Because of the significant difference between the SWE characteristics of hyperplastic parathyroid tissue compared to thyroid tissue, the discriminative diagnostic power of elastography is good; SWE lower than 9.74 kPa identifies parathyroid tissue and not thyroid tissue, with a sensitivity of 94.8%, specificity of 90.7%, and accuracy of 92.26%. We also calculated the SWE ratio parathyroid versus parathyroid and parathyroid versus muscle tissue. The diagnostic qualities were lower than the SWE measurements. Still, a sub-unitary ratio is suggestive for parathyroid tissue, but co-threshold value could be calculated.

There is no threshold value for pathological parathyroid gland size and volume. However, there are multiple data suggesting the correlation between parathyroid diameter and PTH levels [19,46,47], a threshold value of 1 cm being suggestive of a need for medical treatment [47]. Increase in parathyroid volume suggests a descent of sHPT [48]. High correlation between parathyroid SWE and parathyroid size is expectable. 

In our study, we also observed important interrelationship between parathyroid SWE and serum concentrations of PTH, vitamin D, phosphorus, and dialysis performance, Kt/v. Even if there were no significant correlations between the parathyroid volume, serum PTH, vitamin D and phosphorus concentration, and mean SWE elasticity index, we have to keep in mind that tissue elasticity does not depend of size or volume. Elastography measures tissue strain, estimated by finite difference between echo time-delays obtained by cross-correlating echo signals before and after distortion. Hyperplastic parathyroid glands present chief cells dominant in the cell population in early phases of the disease and oncocytes in the advanced cases. Elastography measurement is dependent on the cell occupancy rate and with the phases of the disease.

The main limitation of the study is that we did not have histopathological exams in all patients, but the presence of parathyroid gland enlargement is certified both by ultrasonography and parathyroid scintigraphy. All patients had documented secondary hyperparathyroidism. A proportion of our patients were receiving treatment for secondary hyperparathyroidism during our study. A proportion were receiving treatment with paricalcitol (11 patients), a proportion with cinacalcet (9 patients), and one patient was receiving both treatments. Thirteen patients had previous subtotal parathyroidectomy; the rest of the study group was naive patients. Taking into account all these factors, we could not properly divide the study group into even subgroups.

There are multiple studies cited in the literature that have documented elastographic measurement on parathyroid hyperplasia. However, the measurements are not done in patients admitted in the dialysis centers, on patients with end-stage renal disease. The literature studies have used several elastographic methods and have given multiple cut-off values for parathyroid hyperplasia: 1.46 m/s (determined with shear wave elastography) [29], 1.35 ± 0.61 (elasticity contrast index value and standard deviation determined with Elastoscan Score Index) [49]. The studies included parathyroid hyperplasia, but this were not present on patients with ESRD or on RRT therapy. 

There are multiple research studies performed on ultrasound characteristics of hyperplastic parathyroid glands from secondary hyperparathyroidism that present parathyroid enlargement, with the presence of more than one parathyroid gland, especially in patients following hemodialysis for more than 3 years [50]. The same study states that parathyroid gland increase in size directly proportional to the duration of dialysis. Other studies have not found any correlation between parathyroid hyperplasia and clinical or biochemical features [51].

A larger study performed on 215 parathyroidectomies [52] concluded that renal hyperparathyroidism can be controlled by total parathyroidectomy and forearm graft, but the decision is based on several factors, such as bone disease and vessel calcification. The author also noted that recurrent and persistent hyperparathyroidism is a problem after surgery. Another study stated the ultrasound volume of the enlarged parathyroid glands (larger than 300 mm^3^ or 1 cm in diameter); the likelihood of nodular hyperplasia is present, and the disease may be refractory to medical therapy. Another major problem in the parathyroid surgery is parathyromatosis and parathyroid carcinoma [53]. 

One study concluded that the total time (months) on RRT, especially on hemodialysis, has been associated with increased risk of parathyroidectomy, the strongest association was found to be with the phosphate serum concentration (above 1.85 mmol/L). One independent variable associated with increased risk of parathyroidectomy was the PTH serum concentration [54]. 

Another ultrasound study performed on patients with secondary hyperparathyroidism, with or without RRT therapy, established that ultrasound evaluation should be performed on patients with PTH serum concentrations higher than 400 pg/mL for at last three determinations in 3 months, with or without RRT therapy [48].

Literature studies have concluded that severe vitamin D deficiency in patients with ESRD in hemodialysis RRT, below 12 ng/mL, predicts a high risk of mortality, especially in patients that associate diabetes mellitus [55].

There are clinical implications regarding the use of elastography in diagnosing and treating patients with secondary hyperparathyroidism. It is a simple, operator-independent, repeatable, and reproducible method, complementary to conventional ultrasound, distinguishing between thyroid and muscle tissue. Elastography could bring important information regarding parathyroid elasticity and could be a useful tool in studying and treating secondary hyperparathyroidism.

Other studies in the field could help establish the value of parathyroid elastography in secondary hyperparathyroidism.

## 5. Conclusions

To conclude, the aim of this prospective study was to quantify the value of 2 D shear wave elastography in diagnosing and treating secondary hyperparathyroidism. Elastography can be a useful qualitative and quantitative tool and can offer a better differentiation on tissue elasticity when diagnosing parathyroid hyperplasia from secondary hyperparathyroidism. By using this elastographic technique, a value less than 9.98 kPa for the mean elasticity index is suggestive for parathyroid tissue in patients with secondary hyperparathyroidism.

## Figures and Tables

**Table 1:** Baseline characteristics of the studied group (59 cases with secondary hyperparathyroidism).

Characteristics	Study Group	UCL	LCL
Male to female	27:32	/	/
Dialysis years	5.94 ± 4.49	5.042	6.821
Patients with previous parathyroidectomy	6	/	/
Total number of parathyroid glands	97	/	/
Age (years)	56.95 ± 10.92	54.95	58.97
Parathyroid volume (mL)	0.2199 ± 0.2654	0.1705	0.2764
Parathormone (pg/mL)	1161.86 ± 797.66	979.520	1301.35
Total serum calcium (mg/dL)	8.73 ± 0.76	8.575	8.898
25-OH Vitamin D (ng/mL)	33.57 ± 13.30	30.962	36.076
Kt/v ratio	1.363 ± 0.123	1.3395	1.3865
Phosphorus (mg/dl)	6.313 ± 1.723	5.972	6.664

UCL—upper confidence level; LCL—lower confidence level.

**Table 2:** Two-dimensional shear wave elastography (2 D-SWE) results: Mean value ± standard deviation (SD), minimum value ± SD, maximum value ± SD (results of 97 parathyroid hyperplastic glands).

	Mean SWE (kPa)	95% Confidence Interval		Min SWE (kPa)	Max SWE (kPa)
		Lower Bound	Upper Bound		
Parathyroid gland	7.835 ± 2.944	7.296	8.374	4.630 ± 2.272	12.956 ± 6.126
Thyroid tissue	13.780 ± 4.039	13.112	14.449	9.438 ± 3.606	20.349 ± 7.509
Muscle tissue	15.788 ± 4.409	11.921	13.014	13.851 ± 4.024	23.181 ± 10.493

**Table 3:** Sensitivity, specificity, AUROC for measured SWE-min, SWE-max and SWE-mean.

	SWE-min PTX/T	SWE-mean PTX/T	SWE-max PTX/T	SWE-min PTX/M	SWE-mean PTX/M	SWE-max PTX/M
Area under curve (AUC) value	0.943	0.940	0.858	0.957	0.949	0.882
Specificity	86.6%	90.7%	75.3%	95.9%	90.7%	84.5%
Sensitivity	94.8%	94.8%	83.5%	86.6%	93.8%	78.4%
PPV	87.6%	91.1%	77.1%	95.9%	91%	83.5%
NPV	94.4%	94.6%	82.0%	87.7%	93.6%	79.6%
Accuracy	90.7%	92.26%	79.4%	91.2%	91.75%	81.45%
Cut-off value	<6.02 kPa	<9.74 kPa	<15.3 kPa	<7.94 kPa	<9.98 kPa	<17.3 kPa

**Table 4:** The 2 D-SWE ratio results: Mean value ± SD, minimum value, and maximum value.

SWE Ratio	Mean	Min	Max
Parathyroid/thyroid ratio	0.599 ± 0.250	0.1	1.7
Parathyroid/muscle ratio	0.535 ± 0.273	0.11	1.9

## Abbreviations

ARFI	acoustic radiation force impulse
CKD	chronic kidney disease
CKD-MBD	chronic kidney disease–mineral bone disorder
EI	elasticity index
ESRD	end-stage renal disease
KDIGO	Kidney Disease: Improving Global Outcomes Guidelines
PTH	parathormone
Q-box	quantification box
ROI	region of interest
RRT	renal replacement therapy
sHPT	secondary hyperparathyroidism
SWE	shear wave elastography
SWE-max	maximum stiffness value
SWE-mean	mean stiffness value
SWE-min	minimum stiffness value
SWR	shear wave ratio
